# Different Anthocyanin Profiles of the Skin and the Pulp of Yan73 (Muscat Hamburg × Alicante Bouschet) Grape Berries

**DOI:** 10.3390/molecules15031141

**Published:** 2010-03-02

**Authors:** Jian-Jun He, Yan-Xia Liu, Qiu-Hong Pan, Xiang-Yun Cui, Chang-Qing Duan

**Affiliations:** Centre for Viticulture and Enology, College of Food Science & Nutritional Engineering, China Agricultural University, Beijing 100083, China; E-Mails: hejj.email@gmail.com (J.-J.H.).; yanxia_liu@foxmail.com (Y.-X.L.); panqh@cau.edu.cn (Q.-H.P.); cuixiangyuncxy@126.com (X.-Y.C.)

**Keywords:** teinturier grape, anthocyanin, profile, skin, pulp

## Abstract

Yan73 is a “teinturier” red wine variety cultivated in China and used in winemaking to strengthen red wine color. Here, the anthocyanin profile in both the skin and pulp of this grape variety was analyzed by HPLC-MS. The results showed that 18 anthocyanins were detected in both the skin and the pulp, and pelargonidin-3-*O*-glucoside, an anthocyanin compound hardly detected in most other *Vitis vinifera* berries, was found. However, the contents of individual anthocyanins in the skin and the pulp were significantly different. Compared with the skin, the pulp exhibited much lower ratio of 3’,5’-substituted to 3’-substituted anthocyanins and much higher ratio of methoxylation of anthocyanin B-ring to non methoxylation, and with regard to the aromatic acylated and aliphatic acylated anthocyanins, both their contents in the skin are higher than in the pulp. The findings will provide some new insight for the tissue-specific expression and regulation of the genes involving in anthocyanin biosynthesis in grape berries.

## 1. Introduction

Yan73 (*Vitis vinifera*) is a red wine variety cultivated in China. It was obtained from the hybridization of Muscat Hamburg (*Vitis vinifera* L.) × Alicante Bouschet (*Vitis vinifera* L.) in 1966. Unlike most other grape varieties, Yan73 accumulates pigments not only in the skin, but also in the pulp, so it is commonly used for blending with pale red wine to produce wine of very dense color. The grapes with this property are called “teinturier” or “dyers” [1].

Grape berries contain three major tissue types: skin, pulp and seed, and these tissues contribute differently to overall wine composition. In general, the skin provides volatile and nonvolatile and color compounds, the pulp contribute organic acids and sugars, and the seed provides condensed tannins, all of which are important to the formation of organoleptic characteristics of wine. In terms of most red wine varieties, color compounds almost exist only in the skins, whereas few are in the pulps. The study of Grimplet *et al*. [2] also indicated that some key genes involved in anthocyanin biosynthesis such as the anthocyandin synthase (ANS) gene, UDP-glucose: flavonoid 3-*O*-glucosyltransferase (UDPG) geneand *O*-methyltransferase gene were expressed predominantly in the skin of grape berries. Although a lot of research has described anthocyanin profiles in various red grape varieties [3,4,5], only a few reports have focused on a dyer variety. Ageorges *et al*. [6] found that the anthocyanin profiles in the skin and the pulp of Lacryma dyer variety were very similar, with the exception of peonidin-3-*O*-glucoside and malvidin-3-*O*-(6-*O*-coumaroyl)-glucoside, which were present at higher and lower percentages in the pulp than in the skin, respectively. Balík *et al*. [7] compared the total contents of pigments in some grapes originating from South Moravia, and found the highest level in the variety Neronet (2.15- 4.49 g/kg of fresh grapes), which belongs to the so-called teinturier varieties. Castillo-Muñoz *et al*. [8] studied the anthocyanin composition of Garnacha Tintorera (also known as Alicante Bouschet), and found that malvidin derivatives dominated in skin, followed by peonidin-type anthocyanins, but the flesh almost exclusively contained peonidin-3-*O*-glucoside.

Wine color is one of the most important sensory properties. Therefore, it has long been recognized that the color intensity of young red wines positively correlates, to some extent, with the overall wine quality. Anthocyanins are main pigments responsible for red wine color. According to their structures, they can be classified into the following groups: non-acylated anthocyanins, acylated anthocyanins, pyranoanthocyanins, direct flavanol-anthocyanin condensation products, acetaldehyde-mediated or other compounds-mediated flavanol-anthocyanin condensation products [9,10,11]. But in the skin of red wine grape cultivars (white pulp), the main anthocyanins were five primitive monoglucoside structures and their acetylated or coumaroylated derivatives, most of which are malvidin-3-*O*-glucoside and its acetylated or coumaroylated derivatives. Polymeric anthocyanins, such as pyranoanthocyanins, direct flavanol-anthocyanin condensation products, acetaldehyde-mediated or other compounds-mediated flavanol-anthocyanin condensation products, were hardly detected in grape skins [2,5,12]. The anthocyanins can also be classified according to either the number of hydroxyl groups (3’-substituted anthocyanins and 3’,5’-substituted anthocyanins) or methoxyl groups on B-ring, or the type of acylation (aliphatic or aromatic) [13,14]. The color characteristics of anthocyanins vary with these substituents. Anthocyanins are water-soluble, synthesized in the cytosol of berry cells, and localized in vacuoles. The color of the red wine is essentially due to the release of pigments from the skins of grape berries during the process of winemaking. Therefore, studies of the composition of anthocyanins in the grape berries are helpful for winemaking and quality assessment.

In China, the wines made from Yan73 grape alone do not exhibit fine quality, lacking harmonious and rich body. However, the co-fermentation of Yan73 with other red varieties wines can lead to a significant improvement of wine color. Therefore, Yan73, as a “teinturier” variety, has attracted more and more attention.

For most of the red wine grapes, anthocyanins are mainly located in the three or four layers of cells closest to the outer epidermis, and few in the pulp cells. Until now, the mechanism of specific localization of anthocyanins in the berry tissues remains unclear. For example, such a specific localization is related whether to tissue-specific expression of the genes involving in anthocyanin biosynthesis or to the direct transport of anthocyanin products. In the present study, the anthocyanin profiles in the skin and the pulp of ripening Yan73 berries were investigated at the first time. Yan73, due to pigment accumulation in the pulp, is a rare material for studying tissue-specific expression and regulation of the genes involving in anthocyanin biosynthesis. The present results will provide an essential basis for further research of this field. In addition, these results also will be helpful to find out the possible contribution of the pigment compounds from Yan73 to wine color and potential mechanism of wine coloring. 

## 2. Results and Discussion

**Figure 1:**
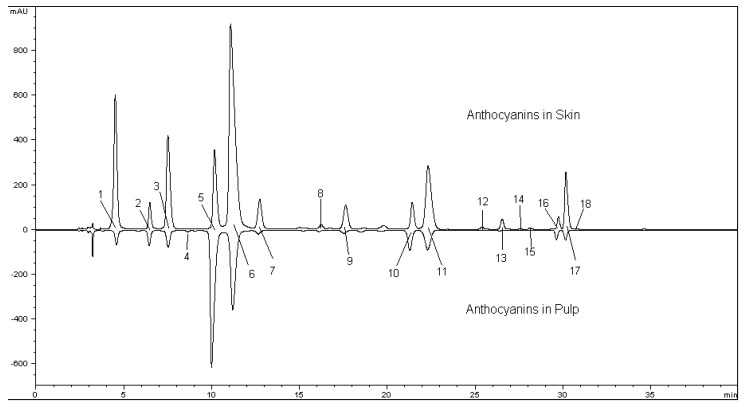
Chromatography of anthocyanins in the pulp and the skin of Yan73 grape. Peak numbers correspond to the labeling adopted in Table 1.

Eighteen anthocyanins were identified from both the skin and the pulp of Yan73, including six primitive anthocyanins and 12 acylated derivatives (Figure 1).

The details of these anthocyanins were listed in Table 1. Total concentration of anthocyanins in the skin was almost equal to that in the pulp, but in terms of individual anthocyanin, there existed significant differences (at 0.05 level, with t-test difference) between the pulp and the skin. Castillo-Muñoz *et al*. detected twenty anthocyanins in Garnacha Tintorera grapes, and they found malvidin derivatives dominated in skin, followed by peonidin-type anthocyanins; but the flesh of Garnacha Tintorera grape almost exclusively contained peonidin-3-*O*-glucoside [8]. In the present study, we also found that malvidin derivatives dominated in the skin of Yan73 grape, which contributed about 67.2% to the total anthocyanins in the skin, but in the pulp, malvidin derivatives and peonidin derivatives contributed 39.0% and 53.1% to total anthocyanins respectively. 

**Figure 2:**
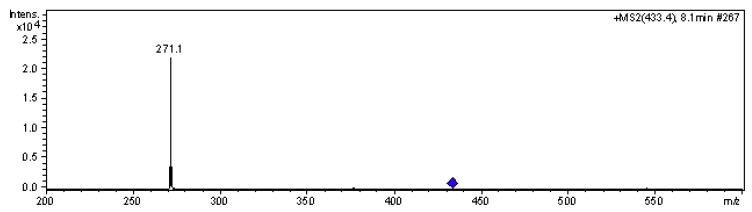
MS2 fragmentation of pelargonidin-3-*O*-glucoside (peak 4).

Interestingly, pelargonidin 3-*O*-glucoside, a primitive anthocyanin compound that is hardly detected in many wine varieties of *Vitis vinifera* [3,6,12], was detected in both the skin and the pulp of Yan73. Pelargonidin- 3-*O*-glucoside was determined according to the following evidence: on the one hand, peak 4 at 7.9 min eluted after petunidin-3-*O*-glucoside (peak 3) that had two hydroxyl group at positions 4’ and 5’ and one methoxyl group at position 3’ on the B-ring, but before peonidin-3-*O*-glucoside (peak 5 - one hydroxyl group and one methoxyl group on the B-ring), which indicated that the polarity of the compound corresponding to peak 4 was higher than that of peonidin-3-*O*-glucoside, but smaller than that of petunidin-3-*O*-glucoside. It has been demonstrated that the introduction of hydroxyl groups will increase the polarity of a compound and of methoxyl groups will decrease the polarity. Therefore, it was first speculated that this compound may have only one hydroxyl group and no methoxyl groups on the B-ring. On the other hand, MS analysis showed that this compound had the molecular ion ([M]+1) at *m/z* 433 and a fragment ion at *m/z* 271 (Figure 2). According to our established standard phenolic library and references [8,15], the *m/z* 271 fragment ion corresponds to the pelargonidin aglycone, and the difference between the ratio of mass to charge (*m/z*) of the molecular ion and fragment ion was 271 (433-162), which corresponded to a hexose, that in grape would mostly be glucose. That is, the compound giving peak 4 lost a glucose fragment ion in the mass spectrum analysis process. Based on the above two points and references [8,15], the compound corresponding to the peak 4 was identified as pelargonidin 3-*O*-glucoside. Pelargonidin 3-*O*-glucoside could be generated from dihydrokaempferol through a catalysis of dihydroflavonol 4-reductase (DFR), although in some plant species such as petunia (*Petunia hybrida*) and cymbidium (*Cymbidium hybrida*), DFR was found to have strict substrate specificity and cannot utilize dihydrokaempferol [16], which may be main reason that in most of *Vitis vinifera* berries pelargonidin 3-*O*-glucoside has not been detected. In the present study, both the skin and the pulp of Yan 73 were detected a little amount of pelargonidin 3-*O*-glucoside, suggesting that there might exist an isozyme of DFR using dihydrokaempferol as substrate to synthesize pelargonidin 3-*O*-glucoside. 

### 2.1. 3’-Substituted anthocyanins and 3’,5’-substituted anthocyanins

**Figure 3:**
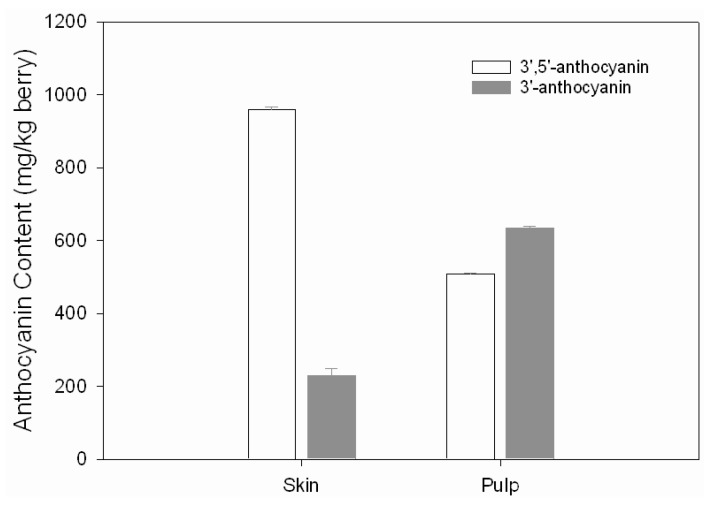
Total concentrations of 3’,5’-substituted anthocyanin and 3’-substituted anthocyanins in the skin and the pulp of Yan73.

According to the number of substituents on the B-ring of the anthocyanin, the latter can be divided into two groups: the group being 3’-substitutents and the other being 3’,5’-substituted, The former includes cyanidin and peonidin monoglycosides and their acylated derivatives, which are called 3’-substituted anthocyanins, and the latter is made up of glycosylated forms of delphinidin, petunidin and malvidin and their acylated derivatives, which are called 3’,5’-substituted anthocyanins [13,14]. Pelargonidin-3-O-glucoside is not considered for it has not any substituents at position 3’ and position 5’ of the B-ring. In ripe Yan73 berries, the total concentration of 3’,5’-substituted anthocyanins (958.73 mg/kg) was over 4-fold higher than that of 3’-substituted ones (229.15 mg/kg) in the skin, but only about 0.8-fold in the pulp (Figure 3). The color of pelargonidin-3-O-glucoside, cyanidin-3-O-glucoside and delphinidin-3-O-glucoside greatly depends on the number of hydroxyl groups on the B-ring. The maximum absorbance wavelengths of pelargonidin-3-O-glucoside (one hydroxyl group on the B-ring), cyanidin-3-O-glucoside (two hydroxyl groups on the B-ring) and delphinidin-3-O-glucoside (three hydroxyl groups on the B-ring) are 504, 516 and 525 nm, respectively, and they tend to give brick red/scarlet, red/magenta, and violet/blue colorations, respectively. The more the number of hydroxyl groups, the stronger the blue hue. In the pulp and skin of Yan73, significant difference (at 0.05 level, with t-test difference) in the ratio of 3’,5’- substituted to 3’-substituted anthocyanins indicated that these two parts might contribute different chromatic characteristics to grape and wine.

The biosynthetic pathways leading to the formation of 3’- and 3’,5’-substituted anthocyanins in grapes are different, the former coming from dihydroquercetin and the latter from dihydromyricetin. Tanaka *et al*. [16,17] found that the transcriptional levels of flavonoid 3’-hyroxylase (F3’-H) and flavonoid 3’,5’-hydroxylase (F3’,5’-H) determined the ratio of 3’-substituted anthocyanins to 3’,5’-substituted anthocyanins, suggesting that the biosynthesis of 3’-substituted anthocyanins might be mainly involved in F3’-H-mediated pathways and the biosynthesis of F3’,5’-H might be mainly involved in F3’,5’-H -mediated pathways. In Lacryma “teinturier” variety, the higher expression of the genes encoded anthocyanin biosynthetic enzymes such as PAL(CX127428), CHS3(CXl26991), F3H1(CX127413), Vv-MybA1(AB097923) and GST (CX12741 1) was shown in the pulp when compared with that in the skin, but the expression of these genes was hardly detected in the pulp of non-“teinturier” variety Gamay [6,16,18], so it is thought that the accumulation of anthocyanins in the pulp of “teinturier” variety should be mainly attributed to the tissue-specific expression of these genes. In the present study, there was the high ratio of 3’,5’-substituted to 3’-substituted anthocyanins in the skin of Yan73, which indicated that the expression level of gene encoding flavonoid 3’,5’- hydroxylase was significantly higher than that of gene encoding flavonoid 3’- hydroxylase in this part of the berry. 

### 2.2. Methoxylated anthocyanins

Methoxylation on the positions 3’ and 5’ of the anthocyanin B-ring could reduce the chemical reactivity of a phenolic hydroxyl group [19]; this is supposed to play an important role in the transformation of the reactive hydroxyl groups of flavonoids [20]. Methoxylation on the position 3’ and 5’ of the anthocyanin B-ring has a slight reddening effect on the color of anthocyanins. [13,14]. Anthocyanins methoxylated on the B-ring include petunidin-3-*O*-glucoside, peonidin-3-*O*-glucoside and malvidin-3-*O*-glucoside. 

**Figure 4:**
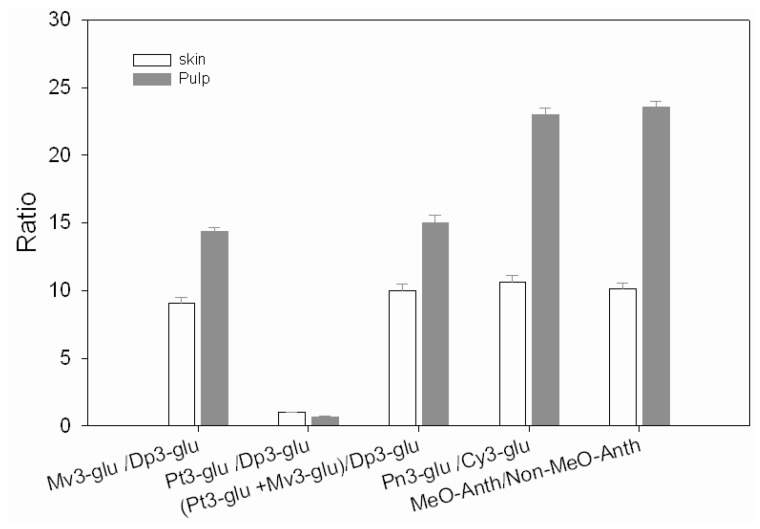
The ratio of methoxylated anthocyanins to non-methoxylated anthocyanins in the skin and the pulp of Yan 73. **Abbreviations:** Dp3-glu for delphinidin-3-*O*-glucoside, Cy3-glu for cyanidin-3-*O*-glucoside, Pt3-glu for petunidin-3-*O*-glucoside, Pn3-glu for peonidin-3-*O*-glucoside, Mv3-glu for malvidin-3-*O*-glucoside, Me*O*-Anth for methoxylated anthocyanins, Non-Me*O*-Anth for non-methoxylated anthocyanins.

Figure 4 shows that the ratios of methoxylated anthocyanins to non-methoxylated anthocyanins, which were 10.14 and 23.55, respectively, in the skin and the pulp of Yan73. Such a big difference (at 0.05 level, the difference is significantly different with t-test difference) in the percentage of methoxylated anthocyanins between the pulp and skin might be related to the tissue-specific expression of the genes encoding *O*-methyltransferases (OMTs) in grape berries. In plants, methoxylation is generally known to be mediated by two distinct classes of *O*-methyltransferases: the first class contains low molecular weight proteins (23,000–25,000), whereas the second includes OMTs of higher molecular weight (38,000–43,000) which use caffeic acid, flavonoid, alkaloids, coumarins, other phenolic derivatives and sugars as substrates [17,18]. OMTs catalyze the methoxylation of 3’-OH of cyanidin-3-*O*-glucoside to generate peonidin-3-*O*-glucoside, and the methoxylation of 3’-OH of delphinidin-3-*O*-glucoside to form petunidin-3-*O*-glucoside, as well as the methoxylation of two hydroxyl groups on positions 3’ and 5’ of delphinidin-3-*O*-glucoside to form malvidin-3-*O*-glucoside. Also, petunidin-3-*O*-glucoside is found from delphinidin-3-*O*-glucoside and then transformed into malvidin-3-*O*-glucoside by two similar reactions induced by a *O*-methyltransferase [17,21,22]. In addition, some other reports showed that malvidin-3-*O*-glucoside is obtained directly from delphinidin-3-*O*-glucoside by the action of *O*-methyltransferases [13]. In Yan73, the ratio of peonidin-3-*O*-glucoside to cyanidin-3-*O*-glucoside is 23.02 in the pulp, much higher than the value 10.63 in the skin, also the ratio (petunidin-3-*O*-glucoside + malvidin-3-*O*-glucoside)/delphinidin-3-*O*-glucoside in the pulp is bigger than in the skin. The ratio of petunidin-3-*O*-glucoside to delphinidin-3-*O*-glucoside is 0.99 and 0.64 in the skin and pulp, respectively, but the ratio of malvidin-3-*O*-glucoside to delphinidin-3-*O*-glucoside is 9.03 and 14.34 in the skin and pulp, respectively. It is thus deduced that delphinidin-3-*O*-glucoside mainly transferred into malvidin-3-*O*-glucoside, only a little transferred into petunidin-3-*O*-glucoside in Yan73 berries. These above results were in agreement with those of Ageorges *et al*. [6], who thought that methoxylation of delphinidin-3-*O*-glucoside B-ring rarely occurred only at the 3′ position. Most frequently, delphinidin-3-*O*-glucoside was methoxylated at both 3′ and 5′ positions, which led to a significant increase of malvidin-3-*O*-glucoside. 

### 2.3. Acylated anthocyanins

**Figure 5:**
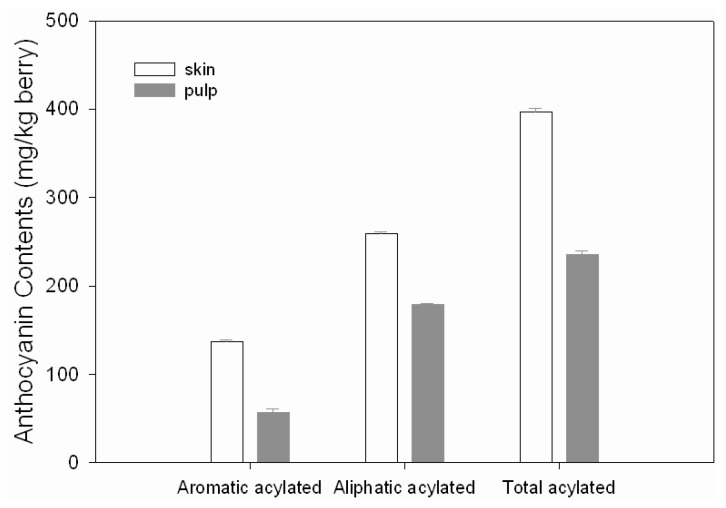
Acylated anthocyanins and non-acylated anthocyanins in the skin and the pulp of Yan73.

Anthocyanins mostly exist as their acylated forms in many plant species. Two major types of acyl substituents of anthocyanins exist, i.e. aromatic (hydroxycinnamic or hydroxybenzoic) and aliphatic (malonic, acetic, or succinic) acyl groups, both of which are commonly linked to 6-OH of glycosyl moiety of anthocyanins. In Yan73 berries, as shown in Figure 5, the level of aliphatic acylated anthocyanins was higher (about double) than that of aromatic acylated anthocyanins in both the skin and the pulp, and as regard to the aromatic acylated and aliphatic acylated anthocyanins, both their contents in the skin are higher than in the pulp.

Anthocyanin acyltransferases (AATs) can catalyze the corresponding acyl transfer from acyl-CoA to the glycosyl moiety of anthocyanins. Aliphatic acyltransferases do not act on aromatic acyl-CoAs, such as p-coumaroyl-CoA and caffeoyl-CoA, and *vice versa* [23,24,25]. It has been proposed that aromatic acylation makes anthocyanins more stable and bluer by intramolecular stacking of the anthocyanins with polyphenols and that the aliphatic acylation of anthocyanin, such as malonylation, does not change the color but is important for enhancing the pigment solubility in water [24], protecting glycosides from enzymatic degradation, stabilizing anthocyanin structures [26,27], or uptaking anthocyanins into vacuoles [28]. Furthermore, anthocyanin acylation should be of nutritional and biomedical importance because some of the bioactivities of anthocyanins have been shown to be strongly modulated by acylation [29]. Therefore, it is suggested that the difference in the ratio of aliphatic acylated to aromatic acylated anthocyanins in between the pulp and the skin might be caused by the higher transcriptional level of AATs or the higher concentration of the aliphatic and the aromatic Co-A in the skin than in the pulp. We can also deduce that the transcriptional level of aliphatic acyltransferases was higher than aromatic acyltransferases, or the contents of aliphatic Co-A substrates were higher than the aromatic Co-A substrates in both the skin and the pulp.

## 3. Experimental

### 3.1. Materials

Yan73 (*Vitis vinifera*) grape berries at commercial maturity were collected in 2006 from the vine base of Penglai, Yantai city, Shandong Province. To obtain a sample representing a vineyard population, we sampled according to the method described by Boulton *et al* [30]. Three 100-berry samples were selected from at least seven 10-clusters at similar position of 30 whole vine selections. These berry samples were carried back to the laboratory. The skin, pulp and seeds were separated by hand immediately, frozen in liquid nitrogen, ground to powder and used for subsequent extraction. 

### 3.2. Chemicals

Acetonitrile, methanol and formic acid (both HPLC grade) were purchased from Fisher (Fairlawn, NJ, USA). Deionized water (<18MΩ resistivity) was obtained from a Milli-Q Element water purification system (Millipore, Bedford, MA). Malvidin-3-*O*-galactoside-chloride, keracoyanidin-chloride, kuromain, malvin chloride, cyanin chloride, pelargonin chloride, delphinidin-3-*O*-glucoside chloride, pelargonidin-3-*O*-glucoside chloride, cyanidin-3-glucoside chloride, peonidin-3-*O*-glucoside chloride, and malvidin-3-glucoside chloride standards was purchased from Extrasynthese SA (Genay, France).

### 3.3. Extraction of anthocyanins

Skin powder or pulp powder (1 g) were extracted in methanol solution (20 mL) containing 1% formic acid. This extraction was performed with the aid of ultrasonics for 10 min, and then shaking in the dark at 25 °C for 30 min at a rate of 150 rpm. The homogenate was centrifuged at 8,000 ×*g* for 20 min and the supernatant was collected. The residues were re-extracted four times, and all the supernatants were pooled into a distilling flask. The methanol solution was removed through a rotary evaporator and the residues were dissolved in white wine (10 mL). Three independent extractions were carried out for either the skin or the pulp.

### 3.4. Qualitative and quantitative analyses of anthocyanins by HPLC-MS

The extracts obtained above were filtered through 0.45 μm filters (cellulose acetate and nitrocellulose, CAN) and the resulting filtrates were directly used for qualitative and quantitative analyses. Each extract was duplicated. An Agilent 1100 series LC-MSD trap VL, equipped with a DAD detector and reversed phase column (Kromasil C18, 250 × 4.6, 5 μm), was used. The solvents were: (A) aqueous 2% formic acid, and (B) acetonitrile containing 2% formic acid. The gradient was from 6% to 10% B for 4 min, from 10% to 25% B for 8 min, isocratic 25% B for 1 min, from 25% to 40% for 7 min, from 40% to 60% for 15 min, from 60% to 100% for 5 min, from 100% to 6% for 5 min, at a flow rate of 1.0 mL/min. Injection volumes were 30 μL, and the detection wavelength was 525 nm. MS conditions were as follows: Electrospray ionization (ESI) interface, positive ion model, 35 psi nebulizer pressure, 10 mL/min dry gas flow rate, 350 ºC dry gas temperature, and scans at m/z 100–1000. The anthocyanins were identified by their order of elution and retention time with respect to malvidin-3-glucoside and the weight of molecular ion and the fragment ion compared with standards and references [31,32,33,34,35,36,37]. The *cis* and *trans* isomers of the coumaroylates for peonidin-3-*O*-glucoside and malvidin-3-*O*-glucoside were distinguished by their elution times and proportions. The *cis* isomers always eluted first on a reverse phase HPLC column and were present in lower proportions than the *trans* isomers in grapes and wines according to previously reported data [35,36,37].

The concentration of all anthocyanins was expressed as malvidin-3-*O*-glucoside. Considering that a great difference in water content in between the skin and the pulp will lead to invalid comparison of anthocyanin contents, the concentration of each anthocyanin was calculated on the basis of fresh weight of the whole berry instead of that of the skin or the pulp. 

### 3.5. Statistical analysis

Origin 8.0 was used to test the differences of the relative amount of anthocyanins in between the skin and the pulp (t-test was carried out).

## 4. Conclusions

In conclusion, the types and total contents of the detectable anthocyanins were similar in the skin and the pulp of Yan73, but the levels of each type of anthocyanins showed significant differences between the skin and the pulp. Pelargonidin-3-*O*-glucoside, which was hardly detected in many wine varieties of *Vitis vinifera*, was detected in both the pulp and skin of Yan73. It is thus suggested that a differential expression of the genes involving in anthocyanin biosynthesis is present in the skin and the pulp of Yan73.These results will also provide some new insights and thus attract us to further study on the anthocyanin biosynthesis and control in grape berries. For example, it needs to be determined what factor to and how to control the expression of the anthocyanin-related genes in the pulp and skin of grape berries, as well as what molecular mechanism to mediate such a differentially tissue-specific expression of the genes in between non-dyer and dyer varieties. Besides, due to specific components of anthocyanins in the pulp, the application of Yan73, as a main dyers variety, in winemaking may profoundly affect the color behavior and stability of wine, which is of interest for future study in the future.

## Figures and Tables

**Table 1:** Anthocyanins detected in the skin and pulp of Yan73 grape berries.

Peak No	Compound	Antho In Skin /Fresh Berry (mg/kg)	Antho In Pulp /Fresh Berry (mg/kg)	λ_max_ (nm)	M^+^ & MS2 (m/z)
1	Delphinidin-3-*O*-glucoside	56.63 ± 3.06	22.54 ± 3.57	524	465, 303
2	Cyanidin-3-*O*-glucoside	14.11 ± 0.89	22.26 ± 0.94	516	449, 287
3	Petunidin-3-*O*-glucoside	56.09 ± 1.57	19.61 ± 2.29	524	479, 317
4	Pelargonidin-3-*O*-glucoside	2.06 ± 0.62	4.79 ± 0.42	504	433, 271
5	Peonidin-3-*O*-glucoside	149.91 ± 2.09	512.33 ± 11.97	518	463, 301
6	Malvidin-3-*O*-glucoside	511.61 ± 3.46	323.12 ± 17.32	528	493, 331
7	Delphinidin-3-*O*-(6-*O*-acetyl-glucoside)	19.25 ± 1.86	7.3 ± 1.35	526	507, 303
8	Cyanidin-3-*O*-(6-*O*-acetyl-glucoside)	1.38 ± 0.06	5.40 ± 0.53	522	491, 287
9	Petunidin-3-*O*-(6-*O*-acetyl-glucoside)	19.92 ± 0.63	6.78 ± 1.46	522	521, 317
10	Peonidin-3-*O*-(6-*O*-acetyl-glucoside)	41.93 ± 0.35	65.82 ± 2.07	522	505, 301
11	Malvidin-3-*O*-(6-*O*-acetyl-glucoside)	176.54 ± 0.71	93.78 ± 5.13	528	535, 331
12	Malvidin-3-*O*-(6-*O*-caffeoyl-glucoside)	2.84 ± 0.05	2.51 ± 0.09	532	655, 331
13	Petunidin-3-*O*-(6-*O*-coumaryl-glucoside)	6.58 ± 0.65	1.98 ± 0.54	530	625, 317
14	Peonidin-3-*O*-(cis-6-*O*-coumaryl-glucoside)	1.18 ± 0.27	1.99 ± 0.17	524	609, 301
15	Malvidin-3-*O*-( cis-6-*O*-coumaryl -glucoside)	4.19 ± 0.58	1.73 ± 0.42	536	639, 331
16	Peonidin-3-*O*-(trans-6-*O*-coumaryl-glucoside)	20.64 ± 0.37	25.95 ± 1.56	522	609, 301
17	Malvidin-3-*O*-( trans-6-*O*-coumaryl-glucoside)	101.77 ± 0.30	22.56 ± 0.64	530	639, 331
18	Malvidin-3-*O*-(6-*O*-feuryl-glucoside)	1.25 ± 0.13	1.98 ± 0.30	532	669, 331
	Total anthocyanins	1,187.88	1,142.43		
